# Curriculum heart failure: Task force curriculum heart failure of the working group on heart failure of the Austrian Cardiological Society

**DOI:** 10.1007/s00508-019-1480-y

**Published:** 2019-05-30

**Authors:** Rudolf Berger, Martin Hülsmann, Deddo Mörtl, Gerhard Pölzl

**Affiliations:** 1 Department of Internal Medicine I, Cardiology and Nephrology, Hospital of St. John of God, Johannes von Gott-Platz 1, 7000 Eisenstadt, Austria; 2 Department of Internal Medicine II, Cardiology, Medical University of Vienna, Vienna, Austria; 3 Department of Internal Medicine 3, University Hospital St. Poelten, Karl Landsteiner Private University, St. Poelten, Austria; 4 University Clinic of Internal Medicine III, Medical University Innsbruck, Innsbruck, Tyrol Austria

**Keywords:** Additional qualification, Training, Specialization, Cardiology, Heart Failure Unit

## Abstract

It is well recognized that organized management of heart failure patients, including care by heart failure specialists, improves outcomes of these patients. In response to this, the Heart Failure Association of the European Society of Cardiology proposed a basic framework of a heart failure curriculum, which became a blueprint for training programs across Europe. The present curriculum for heart failure was well coordinated with the version issued by the German Society for Cardiology in order to achieve good comparability. Training in this Austrian curriculum takes two years, during which the predominant activity focuses on the care of patients with heart failure. The first year includes general (basic) training, while in the second year special modules (advanced chronic and acute heart failure with specific treatment, device treatment, interventional heart failure treatment, outpatient care or rehabilitation, heart failure diagnostics) must be chosen that impart in-depth knowledge, experience and/or skills. Of the five offered modules two must be completed for 6 months each. At least one specialist in internal medicine and cardiology with the additional qualification of heart failure must act as a supervisor at the training center. A certified Heart Failure Unit or a comparable structure should be available at the training center and integrated into the clinical routine of the cardiology department. Applications for recognition of curricular achievements in order to obtain the additional qualification “heart failure specialist” shall be evaluated by a dedicated committee of the nucleus of the Heart Failure Working Group of the Austrian Cardiological Society. The candidate will receive recognition of the additional qualification in heart failure, issued by the Austrian Cardiological Society.

## Preamble

As a result of the enormous advances made in theoretical knowledge and methodologies, cardiology has become increasingly specialized in various areas. The board of the Austrian Cardiological Society (ÖKG) is aware of this development and has commissioned the Working Groups for Heart Failure, Interventional Cardiology, Cardiac Imaging and Rhythmology to draw up curricula for obtaining additional qualifications within cardiology. These curricula are intended to facilitate a further deepening of cardiological knowledge and technical skills within the discipline. On the one hand, they build on the contents of the training in internal medicine and cardiology, while on the other hand they go beyond the theoretical knowledge and technical skills required by the training regulations for cardiology. For these curricula the term additional qualification was chosen in order to distinguish the additional qualification under the umbrella of the scientific societies from the requirement for specializations that are laid down in the Physicians Act (ÄrzteG § 49). Specializations are regulated in the Framework Specialization Ordinance 2015 and in the Specialization Ordinance 2017 of the Austrian Medical Association. An internationally comparable format is explicitly required for a curriculum. The present curriculum for heart failure was therefore coordinated with the version issued by the Heart Failure Association (HFA) of the European Cardiological Society (ECS) [1] and in particular with the versions brought out by the German Society for Cardiology [2] and the Swiss Society for Cardiology [3].

## 1. Introduction

Acute and chronic heart failure are disease patterns that are playing an increasingly important role in cardiology and medicine as a whole. The clinical syndrome “heart failure” has become the most frequent cause for hospital admission in Austria [4]. At the same time, the spectrum of diagnostic and therapeutic options for heart failure has expanded dramatically over the last two decades. In addition, heart failure, which is increasingly understood as a systemic disease, [5] requires a high degree of interdisciplinarity and expertise in neighboring disciplines (e. g. heart surgery, nephrology). Accordingly, the knowledge, experience and skills of the attending physicians required for state of the art treatment have considerably increased. This development has already been taken into account in many clinics by setting up specialized outpatient clinics and in some cases even heart failure units (HFU) [6].

This curriculum is therefore intended to define a framework within which interested colleagues can acquire in-depth knowledge, practical experience and skills in the field of heart failure and obtain certification as such. In future, every cardiologist should have sufficient knowledge of the diagnosis and treatment of heart failure. Patients with advanced heart failure or rare cardiomyopathies in particular, benefit from treatment by colleagues who have been trained in depth and in a structured manner within the framework of a curriculum. In the interest of future Europewide compatibility, the basic structure of this curriculum is based on a proposal made by the Heart Failure Association of the European Society of Cardiology [1] and the curriculum on heart failure drawn up by the German Society of Cardiology [2]. Other international initiatives, e. g. the Heart Failure Society of America (HFSA), were also discussed and considered in partial aspects [7].

## 2. Aim

The aim of specialization within the curriculum of heart failure is to train physicians in the etiology, pathophysiology, diagnostics and treatment of acute and chronic heart failure in all stages, namely beyond the standard of general cardiologists. In this process, comprehensive knowledge is acquired in diagnostics, drug-conservative and interventional, electrophysiological, “device” treatment and intensive care of heart failure. The curriculum is certified by the ÖKG Heart Failure Working Group on the basis of defined knowledge, practical experience and skills, thus ensuring the quality of this specialization. This concerns both the candidates to be trained and the training centers.

## 3. Training implementation—duration and structure of the curriculum

**Fig. 1:**
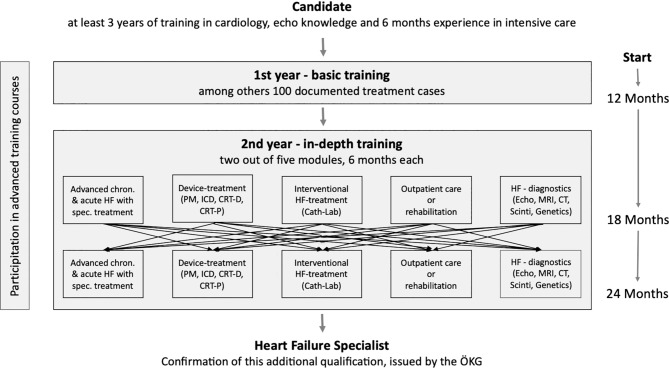
Modular structure of the curriculum heart failure. *CRT-D* cardiac resynchronization therapy defibrillator, *CRT-P* cardiac resynchronization therapy pacemaker, *CT* computer tomography, *Echo* echocardiography, *HF* heart failure, *ICD* implanted cardioverter defibrillator, *MRI* magnetic resonance imaging, *PM* pace maker

Training in the curriculum “heart failure” takes 24 months (2 years), during which the predominant activity focuses on the care of patients with heart failure. According to the proposal made by the Heart Failure Association of the ESC, the first year should include general (basic) training, while in the second year special modules must be chosen that impart in-depth knowledge, experience and/or skills (Fig. 1). Of the five offered modules two must be completed for 6 months each:

Curriculum modules:

- Advanced chronic and acute heart failure with specialized treatment options
- Device treatment for heart failure
- Interventional heart failure treatment
- Outpatient care or rehabilitation
- Specific diagnostics for heart failure

The possibility to choose special modules takes into account the fact that there is no uniform heart failure specialist, as patients are treated at different stages of the disease or in different situations (e. g. outpatient versus inpatient). Some treatment modalities (e. g. interventions for valve diseases) also require expertise that not every heart failure specialist can or must master. At the same time, basic training and proof of having attended pertinent training courses ensure that all heart failure specialists have a comparably high level of knowledge and basic skills. The modules should be completed semester by semester and within 5 years.

## 4. Training contents (detailed list in Appendix)

### 1st year—Basic training

The main aim of the first year of the curriculum is to gain in-depth clinical experience in the diagnosis and treatment of heart failure patients. The candidate should document at least 80 cases using a logbook (e. g. in the form of hospital discharge letters, copies of the patient file). In addition to documentation of the (differential) diagnostic procedure and the systematic recording of comorbidities, the indication for multimodal treatment of heart failure should be mastered (life style interventions, pharmacotherapy, device treatment, interventional treatment, assist procedures, heart transplantation). Each person completing the first year of the curriculum should also have acquired the knowledge and skills to care for patients with acute heart failure and have documented this in at least 20 cases using the logbook. A total of at least 100 patients with acute or chronic heart failure should be diagnosed.

### 2nd year—in-depth training

For specialization in the field of heart failure, the candidate must have completed at least two of the following modules (total training period 12 months Fig. 1). Within the framework of these modules, the candidate should in particular deal with heart failure patients. The required theoretical knowledge must be demonstrated by having completed relevant advanced training courses (see Section 8 “Evaluation and quality control”).

#### Module advanced chronic and acute heart failure with specialized treatment options

In this module, special examination methods, including spiroergometry, biomarkers, myocardial biopsy, as well as a well-founded assessment of hemodynamics, including measurement of pulmonary arterial pressure and resistance, and reversibility testing are to be learned. Furthermore, the indications and differentiated use of pharmacological therapies, mechanical aquaphoretic therapies (ultrafiltration, peritoneal dialysis), as well as ventricular assist devices will be introduced. The selection of patients for heart and heart/lung transplantation and the care of patients before and possibly after heart transplantation will also be taught and learnt.

#### Module device treatment for heart failure

The device treatment module includes implantation and aftercare of implantable cardiac aggregates, e.g. cardiovascular implantable electronic devices (CIEDS), such as antibradycardia pacemakers, implantable cardioverter defibrillators (ICD), cardiac resynchronization therapies defibrillator (CRT-D), and cardiac resynchronization therapies pacemaker (CRT-P) systems.

#### Module interventional heart failure treatment

In this module, the candidate is assigned to the department’s cardiac catheterization laboratory and deals with interventional examinations and therapies in heart failure patients.

#### Module outpatient care (I) or rehabilitation (II)

In this module, the candidate is either (I) trained in the outpatient care of heart failure patients or (II) taught in the rehabilitation of heart failure patients (patient training, treatment optimization, medical training therapy, psychological care).

#### Module specific diagnostics for heart failure

In this module the candidate will receive in-depth training in specific diagnostics related to cardiac imaging (echocardiography, MRI, CT and scintigraphy) and genetic analysis in heart failure patients.

## 5. Candidate requirements

Basic training in internal medicine and cardiology of at least 3 years is required before commencing the curriculum. Moreover, basic echocardiographic knowledge and at least 6 months of intensive care experience are required. Credit may be given for equivalent knowledge, experience and skills in cardiology for a maximum period of 12 months. If all prerequisites are fulfilled, the candidate can be recognized as holding a specialisation in heart failure at the earliest 12 months after completion of training as a specialist in cardiology.

## 6. Requirements for the training center

At least one specialist in internal medicine and cardiology with the additional qualification of heart failure must act as a supervisor at the training center. The supervisor should have 5 years of experience in the care of heart failure patients (see below). Following the position paper heart failure unit (HFU) of the German Society of Cardiology [6] (see below), a certified HFU or a comparable structure should be available at the training center and integrated into the clinical routine of the cardiology department.

### General training

The general training (1st year, 12 months) can be completed at an HFU specialty clinic or a supraregional HFU center.

### Special part

Depending on the modules completed, the special training (2nd year) can be completed at a department with a special HFU clinic, a supraregional HFU center, a special HFU outpatient clinic or a rehabilitation center for cardiovascular diseases with a focus on heart failure. During this period, the candidate is predominantly assigned to the care structures corresponding to the module. To achieve specialization, the candidate must have completed at least two different modules for a total training period of 12 months, namely 6 months for each module (see Fig. 1). Within the framework of these modules, the candidate should in particular be entrusted with the treatment of heart failure patients.

#### Module advanced chronic and acute heart failure with specialized treatment options

This module, in which specialized treatment options, such as heart transplantation and ventricular support systems are introduced, is to be completed at a supraregional HFU center. The center should maintain an active program for heart transplantation and ventricular assist devices or at least be connected to such a center.

#### Module device treatment for heart failure

This module is to be completed at a department with an HFU specialty clinic or a supraregional HFU center.

#### Module interventional heart failure treatment

The module interventional heart failure treatment should be completed in a department with an HFU specialty clinic or a supraregional HFU center. At this center, at least 50 interventional procedures should be performed per year in heart failure patients.

#### Module outpatient care (I) or rehabilitation (II)

The module outpatient care (I) can be completed at a heart failure outpatient clinic of a department with a specialized HFU clinic or a supraregional HFU center or in a specialized HFU outpatient clinic. The rehabilitation module can be completed in an outpatient or inpatient rehabilitation center for cardiovascular diseases with a focus on heart failure.

#### Module specific diagnostics for heart failure

The module specific diagnostics for heart failure should be completed at a department with an HFU specialty clinic or a supraregional HFU center.

Regular conferences with case reviews of heart failure patients should be offered at the center. Instruments for quality assurance (e. g. morbidity and mortality conferences) should also be implemented. The candidate should regularly attend these meetings and have presented at least three cases. The requirements for an HFU in the heart failure network were drawn up on the basis of the corresponding consensus paper issued by the DGK/DGTHG [6]. The following summarizes in brief the most important criteria for the individual HFU modules of the Heart Failure Network.

### HFU outpatient clinic

Staff: cardiology specialist, certified specialist assistant staff (medical assistant or nurse). Cooperation: effective cooperation with an HFU specialty clinic or HFU in the supraregional center. Diagnostics: electrocardiogram (ECG), long-term ECG, stress ECG, possibility for echocardiography and PM, ICD and CRT aftercare, possibility for testing for troponin and natriuretic peptide. Appointments to be given: acute <48 h, post-stationary <7 days.

### HFU speciality clinic (additional features)

Staff at intensive care unit (ICU) or intermediate care unit (IMCU): specialist in cardiology and intensive care medicine or specialist in heart surgery and intensive care medicine, or interdisciplinary management, doctor 24/7 on site, specialist 24/7 on call, key care/patient = 1/4. Cooperation: proven cooperation with HFU in supraregional center. Structure: at least four hospital beds (IMCU or ICU), availability 24/7. Diagnostics: long-term blood pressure monitor, echocardiography (transthoracic and transesophageal), X ray, CT, hemodynamics. Treatment: ventilation (invasive and/or non-invasive), hemodialysis, cardiac catheterization laboratory with 24/7 percutaneous coronary intervention (PCI) availability, ICD/CRT implantation.

### Supraregional HFU center (additional features)

Staff: heart team (cardiologist and heart surgeon) 24/7, ventricular assist device (VAD)/heart transplantation (HTx) coordinator 24/7, if necessary. Structure: at least four hospital beds (IMCU or heart failure ICU) in an area separate from the general intensive care unit, cooperation with heart transplantation or VAD center, if necessary. Diagnostics: sleep apnea screening, lung function test, spiroergometry, cardiac MRI, myocardial biopsy. Treatment: percutaneous cardiac support systems (intra-aortic balloon pump, microaxial pump), extracorporeal life support systems (ECLS), transfemoral aortic valve implantation (TAVI), endovascular mitral valve reconstruction, ablation of complex ventricular tachycardia.

## 7. Training supervisor requirements

The following requirements apply for the training supervisor:

- A physician specialized in internal medicine and cardiology with a concentration on heart failure must act as supervisor at the training institution. The supervisor should have 5 years of experience in the care of heart failure patients.
- This head of the specialization program ensures that the candidates receive the necessary supervision when learning the diagnostic and therapeutic procedures dictated by the curriculum. The specialist also ensures that the candidates attend the formal learning units and courses and are involved in the department’s training and research activities.

## 8. Evaluation and quality control

Evaluation of the candidate with a view to their completion of the curriculum shall consist of the following components:

- Qualification report written by the supervisor of the specialization program. The report contains details on the activities, competence and achieved independence of the candidate. In addition to information on theoretical knowledge and experience, it also contains a description of progress made in practical activities and theoretical knowledge about heart failure.
- Documentation of the patient cases or the examinations/procedures conducted in a logbook (in hard copy or electronic form). The correctness of the logbook is confirmed in writing by the supervisor of the specialization center.
- Documentation of on-going specialist training in the subdiscipline in the form of confirmation of attendance at accredited congresses, workshops, symposia and training/simulation courses held by the specialist societies or their members (HFA meeting, DACH-HF meeting, working group meetings with didactic contents, heart failure consensus meetings). For attendance at a meeting of the major cardiological societies (European Society of Cardiology, American Heart Association, American College of Cardiology) the candidate will be credited a maximum of 2 days. At least five heart failure-specific training days must be documented per training year.

## 9. Accreditation for training

Applications for recognition of curricular achievements in order to obtain the additional qualification “heart failure specialist” shall be evaluated by a dedicated committee of the nucleus of the Heart Failure Working Group of the Austrian Cardiological Society (two co-leaders of the working group, three dedicated members). For this purpose, the documents listed in Section 8 “Evaluation and quality control” are to be submitted. The candidate will receive recognition of the additional qualification in heart failure, issued by the ÖKG. Recognition is valid for 5 years, after which recertification must be applied for (proof of at least one heart failure-specific advanced training per year). Certification as a training institution and certification as a supervisor are granted by the abovementioned committee. The function of supervisor is in principle linked to certification of the training institution. Certification as a training center for the additional qualification is valid for 5 years, after which recertification must be applied for.

## 10. Transitional arrangements

Specialists in internal medicine and cardiology who have been clinically active in the field of heart failure for at least 2 of the last 5 years (including at least two of the required modules) and/or who can demonstrate special scientific expertise in heart failure can acquire the additional qualification “heart failure specialist” on application, without formally completing the curriculum. The applicant’s predominant activity in the area of heart failure as well as the required minimum numbers of the required examinations must be confirmed by the head of the particular institution. The transitional regulation is valid from 1 June 2019 until 30 May 2021.

## Appendix

### Training program schedule

#### 1st year—Basic training

**Table:**

**A) Knowledge**	
Chronic heart failure	
1. Knowledge of the prevention of heart failure	
2. Detailed knowledge of multifactorial etiology and pathophysiology of heart failure	
3. Detailed knowledge of diagnostic examination options for chronic heart failure: a. Cardiac biomarkers a. Imaging (systolic and diastolic function, vitality, ischemia, tissue characterization, etiology—DD of different cardiomyopathies): echocardiography, MRI, nuclear medicine, positron emission tomography (PET), CT, angiography b. Hemodynamics (non-invasive/invasive methods) c. Myocardial biopsy d. Cardiogenetics	
4. Diagnosis and therapy of cardiac arrhythmias in heart failure	
5. Knowledge of cardiorenal syndrome/kidney replacement procedures for chronic heart failure	
6. In-depth knowledge of evidence-based drug-conservative, interventional, and surgical treatment options for acute, chronic, and terminal heart failure	
7. Detailed knowledge of medicinal heart failure treatment and special pharmacotherapy (polypharmacy, interactions)	
8. Knowledge of the specific characteristics and management of the available devices and follow-ups (PM, ICD, CRT, cardiac contractility modulation [CCM], etc.)	
9. Knowledge of the indication for and management of mechanical circulatory support	
10. Knowledge of palliative care for terminal heart failure	
11. Knowledge of structured HI training and training content for patient training	
Acute heart failure (AHF)	
12. Knowledge of etiology and pathophysiology of causes of acute (newly occurring or acutely deteriorated) heart failure (myocardial ischemic/non-ischemic, valvular, pericardial, rhythmogenic, hypertensive, pulmonary vascular, etc.), different forms of presentation of AHF (pulmonary edema, low-output, cardiogenic shock, acute right heart failure in pulmonary embolism, etc.)	
13. Knowledge of the factors triggering AHF	
14. Knowledge of the different acute diagnostic procedures (echocardiography, CT, angiography, scintigraphy)	
15. Knowledge of the different pharmacological therapies for the forms of presentation	
16. Knowledge of guideline-based management of cardiac emergencies (ST-elevation myocardial infarction [STEMI], non-ST-elevation myocardial infarction [NSTEMI], pulmonary embolism, *vitia*, arrhythmias, endocarditis, etc.), including concomitant diseases	
17. Knowledge of surgical and interventional therapy options for different forms of AHF, interdisciplinary decision making/structured consultation in multidisciplinary scenarios (e. g. heart surgeons, pneumologists, intensive care physicians, anesthesiologists, nephrologists, endocrinologists)	
18. Knowledge of the important complications and secondary diseases of AHF (infection, sepsis, multiorgan failure, coagulation disorders, apoplexy, delirium, etc.)	
**B) Experience**	
Chronic heart failure	
1. Indications for specific diagnostic evaluation of heart failure (e. g. echocardiography, MRI, myocardial scintigraphy, myocardial biopsy, coronary angiography, invasive electrophysiological evaluation, genetic counselling/examinations)	
2. Indications for and implementation of medical (conservative) treatment for heart failure	
3. Indications for implantation and follow-up/check-ups for PM, ICD and CRT systems	
4. Indications for interventional therapy of heart failure (PCI, structural interventions, ablations)	
5. Indications for and management of mechanical circulatory support (extracorporeal membrane oxygenation [ECMO] and assist devices)	
6. Indications for renal replacement procedures (dialysis/ultrafiltration) in acute/chronic heart failure	
Acute heart failure	
7. Triage of emergency admission patients with AHF according to clinical risk algorithms	
8. Indications for non-invasive and invasive ventilation, for differential therapy with various renal replacement procedures, for mechanical circulatory support (ECMO, assist devices)	
9. Implementation and interpretation of hemodynamic measurement procedures (e. g. pulse contour cardiac output [PiCCO] catheter, Swan-Ganz catheter)	
10. Acute therapy: resuscitation, non-invasive and invasive ventilation, volume management, pharmacotherapy, ultrafiltration procedures, installation of temporary PM probes	
Overarching learning objectives	
11. Communication with other specialists (healthcare professionals), especially electrophysiologists, interventional cardiologists, imaging specialists (CT, MRI), nephrologists, etc	
12. Work on a heart failure team (including cardiac surgery)	
13. Cooperation between inpatient and outpatient care units	
14. Guidance and care of chronic heart failure patients, their relatives and families	
15. Psychological aspects of patient and family care	
**C) Skills**	**Guiding value**
Chronic heart failure	
1. Care of patients with chronic heart failure	80
2. Echocardiography in patients with chronic heart failure	50
3. Spiroergometry	10
4. Programming pacemakers and ICD/CRT systems	50
5. Right heart catheter (if necessary, including function test)	20
Acute heart failure	
1. Care of patients with acute heart failure according to vital status (e. g. cardiogenic shock, respiratory insufficiency) and guideline algorithms (e. g. acute coronary syndrome, hypertension emergency, arrhythmias, acute mechanical cause [CHAMP] criteria)	20
2. Emergency echocardiography—detection/exclusion of a mechanical cause of AHF	20

#### 2nd year—in-depth training

##### Module: advanced chronic and acute heart failure with specialized treatment options

**Table:**

**A) Knowledge**	
1. Outpatient, inpatient and intensive care of patients with advanced chronic and acute heart failure	
2. Etiology and pathophysiology, right, left and biventricular heart failure, systolic and diastolic dysfunction	
3. Special diagnostics, including spiroergometry, biomarkers, myocardial biopsy	
4. Hemodynamics, including measurement of pulmonary arterial pressure and resistance, reversibility testing	
5. Indication for and differentiated use of pharmacological therapy	
6. Knowledge of mechanical aquapheresis therapies (ultrafiltration, peritoneal dialysis)	
7. Patient selection for heart and heart/lung transplantation listing	
8. Keeping the patient on the heart transplant waiting list	
9. Aftercare following heart transplantation, including immunosuppression and complication management	
10. Patient selection for ventricular support systems, differentiated indication for purely left ventricular and biventricular support systems	
11. Aftercare after implantation of a ventricular support system, including anticoagulation adjustment and complication management	
12. Palliative care concepts	
**B) Experience**	
General information	
1. Intensive care of patients with AHF	
2. Care of patients before and after heart transplantation or implantation of a univentricular or biventricular support system	
3. Right heart catheter examination, including pharmacological reversibility testing of pulmonary arterial and systemic vasoreagibility	
4. Performance and interpretation of spiroergometry	
5. Intravenous therapy with positive inotropic substances, vasopressors and vasodilators	
Patient care before and after heart transplantation (if necessary, by rotation to a dedicated center)	
6. Complete evaluation before heart transplantation	
7. Interdisciplinary indication for heart transplantation listing	
8. Participation in regular heart transplantation conferences	
9. Optional perioperative care for heart transplants	
10. Posttransplantation care and complication management: performance and interpretation of diagnostics in chronic transplant vasculopathy, performance of myocardial biopsy, diagnostics and therapy in humoral or cellular rejection, infections and malignancies	
Patient care before and after implantation of temporary and permanent ventricular support systems	
11. Complete evaluation, including imaging, invasive diagnostics by right heart catheter with vasodilator test and left heart catheterization, myocardial biopsies, spiroergometry, drug therapy	
12. Interdisciplinary indication for left or biventricular support systems	
13. Perioperative care during implantation of a ventricular support system	
14. Postoperative care and complication management for patients with chronic ventricular support systems, including adjustment of pump settings, diagnosis of rhythm disorders, right ventricular failure, bleeding, neurological complications, infections	
**C) Skills**	**Guiding value**
Acute heart failure	
1. Administration and monitoring of therapies with inotropics, vasodilators and vasopressors	50
2. Implantation of central venous and arterial accesses	30
3. Establishment and evaluation of invasive methods for measuring hemodynamics (pulmonary artery catheter, PiCCO, etc.)	20
4. Use of non-invasive and invasive ventilation methods	20
5. Use of mechanical kidney replacement methods for liquid balancing	10
6. Implantation of temporary cardiac support systems (intra-aortic balloon pump [IABP], Impella heart pump, ECMO, etc.)	5
Advanced/terminal heart failure	
7. Outpatient care of patients before and after HTx	20
8. Outpatient care before and after VAD implantation	10
9. Inpatient care of HTx/VAD patients with long-term complications	10

##### Module: device treatment for heart failure

**Table:**

**A) Knowledge**	
1. Selecting suitable patients for ICD and CRT therapy using existing national and international guidelines	
2. Detailed knowledge of electrostimulation, defibrillation, probe and device technology	
3. Detailed knowledge of hemodynamics of electrostimulation, defibrillation and resynchronization	
4. Complication management in the long-term care of patients with ICD and CRT systems	
5. Implantation, explantation and revision techniques, including their complications	
6. Detailed knowledge of the function and programming of ICD and CRT systems as well as their stimulation and defibrillation forms	
7. Telemonitoring (remote monitoring) to detect and prevent exacerbation of heart failure	
8. Diagnostic device functions	
9. Analysis of pacemaker and ICD ECGs and intracardiac electrograms	
10. Detailed knowledge of the interaction between medication and therapy optimization (medication, monitoring, programming)	
11. Legal, ethical and socioeconomic aspects	
**B) Experience**	
1. Interpretation of 12-channel ECGs, 24 h long-term ECGs and other recording systems (i. e. external/implantable loop recorder)	
2. Programming and analysis of memory information in ICD and CRT systems	
3. Detailed experience with probe placement, especially the left ventricular probe in CRT systems (if necessary, alternative probe placement by epicardial electrode implantation)	
4. Identification of non-responders in CRT systems	
5. Optimized drug therapy and maximum biventricular stimulation therapy	
6. Interpretation of intracardiac electrocardiograms (EGM) produced by the devices	
7. Recognition of device problems and their solution	
8. Benefits of echocardiography in program optimization of CRT systems (AV delay, VV delay, pre-ejection period, mitral insufficiency, mitral inflow profile, asynchronicity)	
9. Independent aftercare of all active electrical implants, experience in the use of telemonitoring and patient monitoring, long-term care of patients with ICD and CRT systems	
**C) Skills**	**Guiding value**
1. ICD implantations as primary surgeon (initially under supervision)	25
2. CRT implantations as primary surgeon (initially under supervision)	10
3. Follow-up of ICD systems	50
4. Follow-up of CRT systems	50

##### Module: interventional heart failure treatment

**Table:**

**A) Knowledge**	
1. Hemodynamics in systolic and diastolic cardiac failure and concomitant or sequel diseases, including functional and degenerative mitral and tricuspid valve insufficiency, aortic valve stenosis or insufficiency, ventricular aneurysm, congenital and corrected congenital heart defects	
2. Clinical care before, during and after interventional treatment	
3. Invasive cardiac diagnostics, including left heart catheter examination, right heart catheter examination with reversibility test; if necessary, myocardial biopsy	
4. Periprocedural imaging, including transthoracic and transesophageal echocardiography	
5. Indication for surgical procedures in heart failure, including aortocoronary bypass surgery, valve replacement or reconstruction, ventricular resection plastic surgery	
6. Indication for temporary mechanical circulatory support	
7. Indication for (high-risk) coronary intervention in heart failure, possibly with temporary mechanical circulatory support	
8. Indication for endovascular therapy of aortic, mitral and tricuspid valve diseases	
9. Indication for alcohol septum ablation (ASA) in hypertrophic obstructive cardiomyopathy (HOCM)	
**B) Experience**	
1. Performance and evaluation of left and right heart catheter examination, including reversibility test	
2. Care of cardiac insufficiency patients before and after interventional cardiac insufficiency procedures, including necessary pharmacological treatment	
3. Selection of technique, access route, necessary catheters and instrumentation	
4. Complication management before, during and after interventional cardiac insufficiency procedures, particularly with repect to coagulation, bleeding, thrombosis, allergy, kidney failure and infections	
**C) Skills**	**Guiding value**
1. Performing interventions in patients with symptomatic heart failure: a. Application and care of percutaneous circulatory support systems b. (High-risk) coronary intervention in heart failure, possibly with temporary mechanical circulatory support c. Endovascular valve therapy (mitral/tricuspid valve reconstruction, aortic/central valvuloplasty or replacement) d. Interventional ventricular reduction/VSD occlusion e. Atrial septal interventions (ASD closure) f. Alcohol septum ablation (ASA) in hypertrophic obstructive cardiomyopathy (HOCM)	30

##### Module: outpatient care (I) or rehabilitation (II)

**Outpatient care (I)**

**Table:**

**A) Knowledge**	
1. Long-term coordination of diagnostics and therapy	
2. Survey of anamnesis, symptoms, current status and quality of life	
3. Advice on nutrition and life style: physical activity, heart sports group, nutrition and daily weight control, fitness to drive, fitness to travel, sexual activities	
4. Review and indication for extended medical and interventional/surgical therapy	
5. Verification of adherence and verbal intervention to improve adherence with respect to drug therapy	
6. Consideration of the patient’s preferences in heart failure therapy	
7. Treatment in acute decompensation: outpatient vs. inpatient	
8. Detection and management of non-cardiac concomitant diseases, including mental disorders	
9. Interdisciplinary cooperation for treatment of the underlying disease and associated diseases (family doctor, nephrologist, pneumologist, diabetologist, angiologist, inpatient sector, cardiac insufficiency center, cardiac sports groups, physiotherapy, palliative medicine)	
10. Examination of indication for palliative therapy; if necessary, introduction of palliative therapy	
11. Patient care in a disease management program (DMP)	
**B) Experience**	
1. Evaluation by means of apparatus-based procedures, especially echocardiography (according to indication: ECG, stress ECG, long-term ECG, spiroergometry, stress echocardiography, MRI, invasive diagnostics)	
2. Query and patient-specific programming of implanted cardiac aggregates (CIEDS), including telemedical care	
3. Determination/evaluation of laboratory values (in particular NT-proBNP, renal function, electrolytes, liver values, anemia)	
4. Review and adjustment of current medication (medication according to guidelines, titration, optimal or maximum tolerable dosage, undesirable effects)	
5. Interdisciplinary cooperation in a DMP	
**C) Skills**	**Guiding value**
1. Care of patients in a heart failure outpatient clinic including therapy decisions	200
2. Conducting diagnostic examinations with technical tools (see Experience)	–
3. Active participation in an interdisciplinary DMP	–

**Rehabilitation (II)**

**Table:**

**A) Knowledge**	
1. Survey of anamnesis, symptoms of current status and quality of life and psychosocial aspects	
2. Advice on nutrition and life style: physical activity, nutrition and daily weight control, fitness to drive, fitness to travel, sexual activities	
3. Review and indication for extended drug and interventional/surgical therapy	
4. Verification of adherence and verbal intervention to improve adherence with respect to drug therapy	
5. Consideration of the patient’s preferences in heart failure therapy	
6. Treatment with acute decompensation: outpatient versus inpatient	
7. Detection and management of non-cardiac concomitant diseases, including mental disorders	
8. Medical training therapy (endurance training and strength training)	
9. Patient care in a DMP	
**B) Experience**	
1. Evaluation by means of apparatus-based procedures, especially echocardiography (if necessary, according to indication: ECG, stress ECG, long-term ECG, spiroergometry, stress echocardiography)	
2. Query and patient-specific programming of implanted cardiac aggregates (CIEDS)	
3. Determination/evaluation of laboratory values (especially NT per BNP, renal function, electrolytes, liver values, anemia)	
4. Review and adjustment of current medication (medication according to guidelines, titration, optimal or maximum tolerable dosage, undesirable effects)	
5. Interdisciplinary cooperation in a DMP	
6. Socio-medical assessment for occupational reintegration, job analysis	
7. Driving aptitude	
8. Conception and management of specialized heart groups	
9. Guidance and care of chronic HI patients, their relatives and families	
10. Psychological aspects of patient and family care	
**C) Skills**	**Guiding value**
1. Active participation in patient training	50
2. Definition of a training program for outpatients or inpatients	50
3. Participation in an interdisciplinary psychocardiological program	–
4. Participation in an interdisciplinary disease management program	–
5. Medical training therapy (endurance, strength)	–
6. Care of HTx/VAD patients with complications in postoperative setting and in long-term course	–

##### Module: specific diagnostics for heart failure

**Table:**

**A) Knowledge**	
1. Knowledge of the various imaging techniques used to select the optimal imaging method for identifying the cause and mechanism of heart failure	
2. Knowledge of how to use the full range of common and validated diagnostic tools to determine the nature and severity of heart disease and for clinical management of patients	
3. Knowledge of the phenotype of various etiologies and factors indicating potentially reversible factors	
4. Thorough understanding of the echocardiography techniques and experience in implementing and monitoring the listed modalities	
5. Enhanced understanding of MRI techniques and experience in performing and monitoring the listed modalities	
6. Greater understanding of the scintigraphy techniques and experience in performing and monitoring the listed modalities	
7. Enhanced understanding of cardiomyopathy-specific genetic analyses and experience in their interpretation	
**B) Experience**	
General information	
1. Advanced expertise in the interpretation of echocardiography, cardiac MRI, coronary CT and scintigraphy	
2. Patient selection for CMR, expert echocardiography, scintigraphy, coronary angiography, and coronary CT	
3. Demonstrated assistance in the workup of patients with all etiologies of heart failure	
4. Certification of echocardiography competence (European Association of Echocardiography or national equivalent); ability to independently and competently assess echocardiography and cardiac MRI	
Transthoracic echocardiography in patients with heart failure	
5. Evaluation of the left ventricular and right ventricular systolic function both globally and regionally (if necessary, using contrast medium echocardiography), determination of the left ventricular stroke volume on the basis of the 2‑D LV volumes, calculation of the left ventricular pressure rise rate (dp/dt)	
6. Determination of diastolic function by mitral inflow profile (E/A) or determination of mitral annulus velocity by tissue Doppler (E/é), including indirect parameters such as atrial volumes; knowledge of specific parameters such as pulmonary venous flow velocity (systole/diastolic) and ratio of atrial backflow to duration of A wave	
7. Differentiated and quantitative evaluation of cardiac valve defects by color Doppler echocardiography and CW or PW Doppler; evaluation of cardiac valve dysfunction according to geometric changes in the heart chambers as a result of heart failure (e. g. restriction of the mitral valve, tenting, etc.), determination of pulmonary arterial pressure by CW Doppler in tricuspid valve insufficiency	
8. Diagnosis of ischemia and vitality by means of stress echocardiography	
Transesophageal echocardiography in patients with heart failure	
9. Differentiated evaluation of valve pathologies and heart structures (e. g. atrial septal defect [ASD]), further evaluation of pathological intracardiac structures	
10. Procedural accompaniment of interventions such as interventions on the mitral valve including 3D echocardiography	
Cardiac MRI	
11. Experience in the precise determination of systolic (cardiac output [CO], left ventricular [LV-] and right ventricular [RV-] ejection fraction [EF] etc.) and diastolic heart function, as well as in the determination of volumes and muscle mass	
12. Experience in tissue characterization (e. g. early gadolinium enhancement (EGE), T1/T2 mapping, late gadolinium enhancement (LGE), T2* measurement) and etiology (DD ischemic cardiomyopathy, myocarditis, amyloidosis, sarcoidosis, Chagas disease, Fabry disease, non-compaction cardiomyopathy, or hemochromatosis)	
13. Experience in the diagnosis of ischemia and vitality as well as the diagnosis of *vitia*	
Scintigraphy	
14. Experience in the diagnosis of ischemia and vitality	
15. Experience in the diagnosis of amyloidosis	
Genetic analyses	
16. Indexing and assessing cardiomyopathy-specific (hypertrophic cardiomyopathy [HCMP], idiopathic CMP, arrhythmogenic right ventricular cardiomyopathy [ARVC]) genetic analyses	
**C) Skills**	**Guiding value**
1. Transthoracic echocardiography in patients with heart failure	200
2. Part of these transthoracic echocardiography will include a differentiated analysis of systolic and diastolic function by means of tissue Doppler	50
3. Transesophageal echocardiography in patients with heart failure	25
4. Diagnosis (alone or on the cardiological-radiological team) of cardiac MRI in patients with heart failure	30
5. Diagnosis (alone or on the cardiological-radiological team) of coronary CT in patients with heart failure	30
6. Diagnosis (alone or on the cardiological-nuclear medicine team) of myocardial scintigraphy in patients with heart failure	20
7. Indexing and assessing cardiomyopathy-specific genetic analyses	10
